# The intervals method: a new approach to analyse finite element outputs using multivariate statistics

**DOI:** 10.7717/peerj.3793

**Published:** 2017-10-13

**Authors:** Jordi Marcé-Nogué, Soledad De Esteban-Trivigno, Thomas A. Püschel, Josep Fortuny

**Affiliations:** 1 Centrum für Naturkunde, University of Hamburg, Hamburg, Germany; 2 Virtual Palaeontology, Institut Català de Paleontologia, Bellaterra, Spain; 3 Transmitting Science, Piera, Spain; 4 School of Earth and Environmental Sciences, University of Manchester, Manchester, United Kingdom; 5 Centre de Recherches en Paléobiodiversité et Paléoenvironnements, Museum national d’Histoire naturelle, Paris, France

**Keywords:** Biomechanics, Finite element analysis, Cingulata, Armadillos, Chewing mechanics, Multivariate analysis

## Abstract

**Background:**

In this paper, we propose a new method, named the intervals’ method, to analyse data from finite element models in a comparative multivariate framework. As a case study, several armadillo mandibles are analysed, showing that the proposed method is useful to distinguish and characterise biomechanical differences related to diet/ecomorphology.

**Methods:**

The intervals’ method consists of generating a set of variables, each one defined by an interval of stress values. Each variable is expressed as a percentage of the area of the mandible occupied by those stress values. Afterwards these newly generated variables can be analysed using multivariate methods.

**Results:**

Applying this novel method to the biological case study of whether armadillo mandibles differ according to dietary groups, we show that the intervals’ method is a powerful tool to characterize biomechanical performance and how this relates to different diets. This allows us to positively discriminate between specialist and generalist species.

**Discussion:**

We show that the proposed approach is a useful methodology not affected by the characteristics of the finite element mesh. Additionally, the positive discriminating results obtained when analysing a difficult case study suggest that the proposed method could be a very useful tool for comparative studies in finite element analysis using multivariate statistical approaches.

## Introduction

The introduction of virtual models applied to biological structures represents an important advance for comparative biological studies achieved during the last few years (see review in Bright, 2014). In computational biomechanics, FEA has become increasingly popular among researchers due to its ability to show the biomechanical behaviour of anatomical structures, and is especially useful for analysing species where experimental approaches are not suitable (Gunz et al., 2009; Pierce, Angielczyk & Rayfield, 2009; Degrange et al., 2010; Fletcher, Janis & Rayfield, 2010; Fortuny et al., 2011; Fortuny et al., 2015; Fortuny et al., 2016; Attard et al., 2016; Figueirido et al., 2014). FEA is a technique that acts by dividing a structure into a finite number (normally thousands or millions) of discrete elements with well-known mathematical properties (e.g., triangles, tetrahedrons or cubes). If the geometry of an object is simple enough, strain and stress can be solved by applying analytical solutions. However, more complex shapes (such as the ones observed in most biological cases) might be difficult or even impossible to solve using analytical means, especially if the loading scenarios or material properties are complex. Therefore, FEA offers an alternative approach by approximating the solution via the subdivision of complex geometries into multiple finite elements of simpler geometry. After virtually applying forces to the structure under analysis the stress (and strain) produced by those loads is computed in each one of these small elements.

The results obtained using FEA are commonly expressed as colour maps where warmer colours (i.e., orange, red) represent high levels of stress, whilst colder colours (i.e., blue) correspond to lower levels. These FEA-derived colour maps have proven to be very useful in biological contexts, especially when the main aim of a study is to detect which regions of a particular structure are most affected by the applied loads (Rayfield, 2004).

In spite of the usefulness of these colour maps (e.g., it is possible to define the most fragile area of a structure by visual inspection), they do not easily allow a quantitative performance comparison between similar structures. Comparative approaches in functional morphology focus on elucidating the differences between species (or another taxonomic level) for the same anatomical structure (Neenan et al., 2014). Therefore, their main aim is to test which species are better prepared to bear equivalent loads instead of actually knowing the specific amount of stress/strain that would break the structure under analysis (Lautenschlager, 2017). Researchers are usually looking for the connection between the observed amount of stress in the analysed taxa and some ecologically relevant variable.

When the taxonomic level is not very high (e.g., at the genus or family level), the structure (e.g., a specific bone) is usually quite similar, thus making the visual inspection of the colour maps more difficult or even not conclusive at all (Serrano-Fochs et al., 2015). Usually researchers interpret the colour maps visually and translate that qualitatively (species more “bluish” have less stress than those more “reddish”). Although these descriptions might be useful to provide an overall summary of the results, they are highly subjective and imprecise which makes them problematic to report differences (Dumont et al., 2011).

This problem has been tackled by researchers in the last few years by applying different approaches in order to obtain quantitative data that can be later used to compare different species and to test hypotheses in comparative contexts. One possible approach is to compute the mean of the von Mises stress values of different taxa and then compare them (McHenry et al., 2007; Farke, 2008; Aquilina et al., 2013; Figueirido et al., 2014; Neenan et al., 2014; Fish & Stayton, 2014; Lautenschlager, 2017). However, as described in Bright & Rayfield (2011), Tseng & Flynn (2014), Marcé-Nogué et al. (2015b), Marcé-Nogué et al. (2016) this approach has the problem that part of the observed variation can be produced by the differences in the size of the elements of the finite element meshes representing each taxon. Therefore, some correction is required when computing the mean of the von Mises stress values. For instance, Marcé-Nogué et al. (2016) proposed a method that weights the stress values by the size of the element in order to obtain corrected mean values. Another possible approach is to use box-plots or other visual ways of representing distributions (e.g., histograms) to compare in a general manner whether one taxon shows more stress than another one (Farke, 2008; Figueirido et al., 2014; Fortuny, Marcé-Nogué & Konietzko-Meier, 2017). Finally, another proposed solution is to collect von Misses stress values at specific points or slices to compare the biomechanical performance between different species (Piras et al., 2015; Serrano-Fochs et al., 2015; Püschel & Sellers, 2016; Attard et al., 2016).

Despite the usefulness of all the above-mentioned approaches, they are still only gross measurements that do not make the most of the results obtained from FEA. There is still a need for a quantitative meaningful output from FEA that could be used in multivariate statistical analyses, since most applied multivariate approaches (Marcé-Nogué et al., 2015a; Fortuny et al., 2016) only analyse stress values collected from a limited number of points. Therefore, the main aim of this work is to present a new approach, which we have named the interval’s method, which allows the quantitative comparison of FEA results from different specimens in a multivariate statistical framework.

The second objective of this work is to check whether the proposed intervals method is useful when testing biologically meaningful hypotheses using real data. The proposed method was applied to compare the stress results obtained from several planar models of armadillo mandibles to test the hypothesis that there are significant differences in biomechanical performance (measured as stress values) between different dietary categories.

## Material and Methods

### FEA models

Plane models of 11 mandibles of Cingulata (Table 1), each one corresponding to a different species, were created according to the methodology summarized by Fortuny et al. (2012). The models were created using the ANSYS FEA Package (Ansys Inc.) v.15 for Windows 7 (64-bit system) to obtain the von Mises stress distribution.

**Table 1:** List of the species analysed in the present study. The classification of each species was made on the basis of the current knowledge about the ecology of armadillos, mainly based on stomachs contents (Soibelzon et al., 2007; Redford, 1985; Redford & Wetzel, 1985; Sikes, Heidt & Elrod, 1990; Bolkovi, Caziani & Protomastro, 1995; Smith, 2008; Da Silveira Anacleto, 2007; Superina et al., 2009; Abba et al., 2011; Loughry & McDonough, 2013; Borghi et al., 2011; Superina, Pagnutti & Abba, 2014;  Dalponte & Tavares-Filho, 2004;  Hayssen, 2014;  McBee & Baker, 1982). The geometric properties and the applied forces at the Masseter and Temporalis muscles are also provided. Abbreviations preceding the names of institutions are used to identify the location were the specimens are housed. AMNH, American Museum of Natural History, New York, USA; MNCN, Museo Nacional de Ciencias Naturales, Madrid, Spain; MNHN, Muséum National d’Histoire Naturalle, Paris, France; ZMB, Zoologisches Museum, Berlin, Germany; MLP, Museo de la Plata, La Plata, Argentina.

Taxon	Diet	Collection number	Thickness (mm)	Model area (mm^2^)	Masseter area (mm^2^)	Temporalis area (mm^2^)	Masseter force (N)	Temporalis force (N)
*Priodontes maximus*	Specialist insectivore	AMNH 208104	6.41	2051.70	616.02	255.06	1.29	0.53
*Cabassous unicinctus*	Specialist insectivore	MNHN 1953/457	3.51	415.75	112.08	22.91	0.37	0.08
*Tolypeutes matacus*	Generalist insectivore	AMNH 246460	3.56	497.40	157.01	64116.00	0.35	0.14
*Dasypus kappleri*	Generalist insectivore	MNHN 1995/207	3.51	971.37	105.37	153.18	0.28	0.41
*Dasypus sabanicola*	Generalist insectivore	ZMB_Mam_85899	2.78	527.86	150.66	71545.00	0.27	0.13
*Dasypus novemcinctus*	Generalist insectivore	AMNH 133338	2.94	613.54	225.77	92174.00	0.32	0.13
*Chlamyphorus truncatus*	Generalist insectivore- fossorial	ZMB_Mam_321	2.00	113.19	16035.00	34006.00	0.04	0.09
*Chaetophractus villosus*	Omnivore/ Carnivore	MNCN 2538	4.94	1038.90	300.58	156.08	0.66	0.34
*Chaetophractus vellerosus*	Omnivore/ Carnivore	MLP 18.XI.99.9	3.68	538.80	145.04	117.03	0.30	0.24
*Euphractus sexcinctus*	Omnivore/ Carnivore	MNHN 1917/13	5.66	1019.20	331.22	190.60	0.72	0.41
*Zaedyus pichiy*	Omnivore/ Carnivore	MLP 9.XII.2.10	3.51	327.35	89737.00	66091.00	0.23	0.17

Two main masticatory muscles (i.e., temporalis and masseter) were included in the model as a vector between the centroid of the muscular attachment in the mandible and the centroid of the equivalent muscle attachment in the skull following the modelling approach used in Serrano-Fochs et al. (2015). To compare the models, a scaling of the values of the forces was applied according to a quasi-homothetic transformation in the FEA models (Marcé-Nogué et al., 2013) using the plane model of *Chaetophractus villosus* as a reference. This method corresponds to an adaptation of the scaling methods proposed by Wroe, McHenry & Thomason (2005) and Dumont, Grosse & Slater (2009) for plane models. This procedure was performed to apply the appropriate force in each model, thus allowing the comparison of the stress results when the specimens differ in size.

The information for each analysed species regarding the area of the mandible, insertion areas, forces (musculature applied force per unit area (N/mm^2^)), thickness and the scale factor in the quasi-homothetic transformation can be found in Table 1.

**Figure 1:**
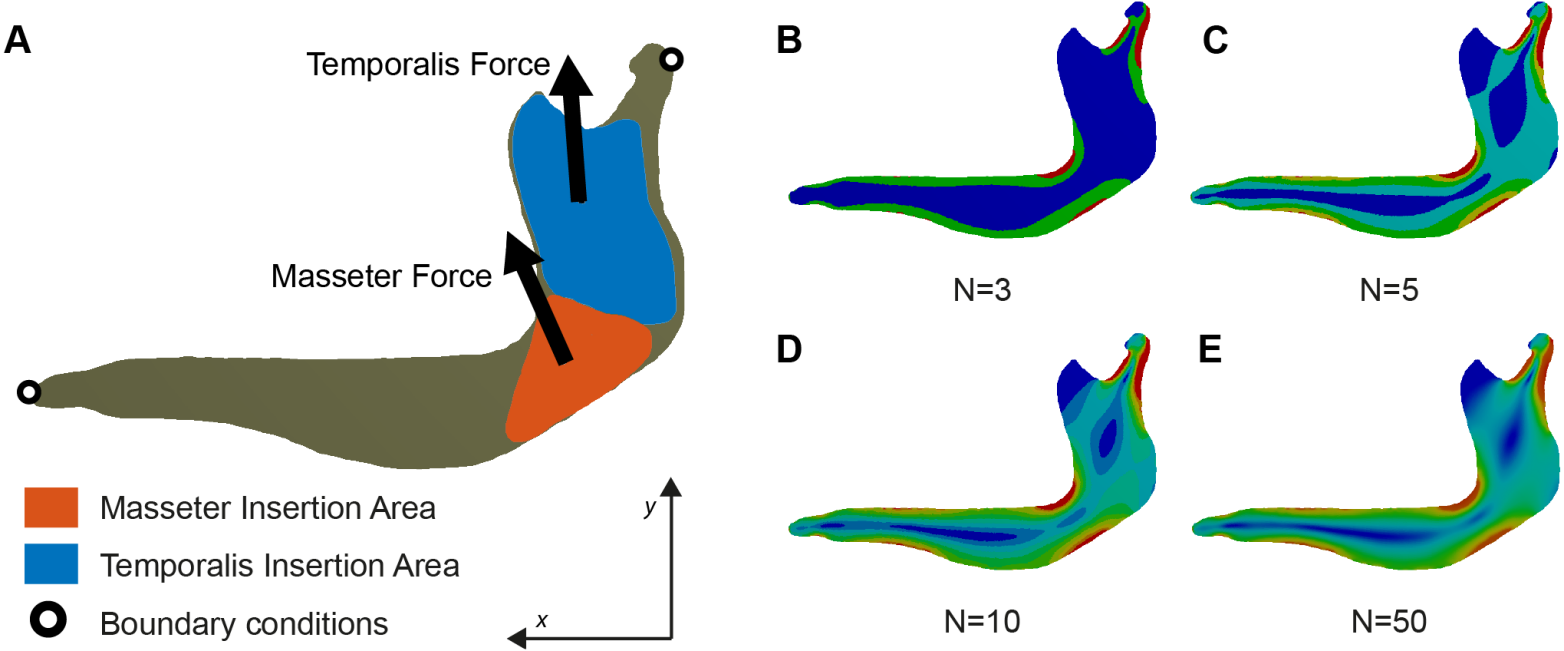
(A) Free-Body diagram of the Biomechanical problem and (B–E) representation of von Mises stress distribution in a mandible of *Chlamyphorus truncatus* with different number of intervals (N) under the same boundary conditions.

The boundary conditions were defined and placed to represent the loads, displacements, and constraining anchors that the structure (i.e., mandible) experiences during its function. The mandible was constrained in the *x* and *y* direction at the most anterior part and fixed in the *x* and *y* directions on the condyle at the level of the mandibular notch (Fig. 1) following the procedures described in Serrano-Fochs et al. (2015) and Marcé-Nogué et al. (2016).

Isotropic and linear elastic properties were assumed for the bone. In the absence of data for Cingulata or any other closer relative, as well as lacking data for any mammalian clade with a similarly shaped jaw, we decided to apply the mandibular material properties of *Macaca rhesus*: *E* (Elasticity Modulus) = 21,000 MPa and *v* (Poisson coefficient) = 0.45 (Dechow & Hylander, 2000). We chose the available properties of *Macaca rhesus* because it has a wide range of habitats and diet which resembles omnivorous or generalist insectivorous armadillos (Richard, Goldstein & Dewar, 1989). In addition, it has been shown that in a comparative analysis these values are not crucial (See Gil, Marcé-Nogué & Sánchez, 2015 for discussion).

As primary data, we obtained the von Mises stress distribution of each one of the analysed species. Von Mises stress is an isotropic criterion used to predict the yielding of ductile materials determining an equivalent state of stress (Reddy, 2008). Considering bone as a ductile material (Dumont, Grosse & Slater, 2009) and according to Doblaré, García & Gómez (2004) when isotropic material properties are defined in cortical bone, the von Mises criterion is the most adequate for comparing stress states.

### Quantitative stress data

Von Mises stress values were obtained for all the elements of each finite element model. Values of stress (and strain) can be obtained for each element because FEA mathematically solves stress (and strain) inside each element, while displacements are computed at the nodes (Zienkiewicz, Taylor & Zhu, 2013). ANSYS provides a complete data file with these results that can be easily manipulated in spreadsheets and other software. This means that the number of stress data points for each mandible is the same as the number of elements of the mesh.

The new methodology proposed here divides the values of stress into N equal intervals (Fig. 1). Therefore, each interval will contain all the elements within a certain threshold, each one being defined by a lower threshold *T*_lower_ and an upper threshold *T*_upper_. The range of the intervals is constant across the sample, the distance from *T*_lower_ to *T*_upper_ being the same in all the intervals. For an interval Φ_*i*_ the lower threshold coincides with the upper threshold of the interval Φ_*i*−1_ and the upper threshold coincides with the lower threshold of the interval Φ_*i*+1_.

Once all the stress values of a single specimen were obtained they were subdivided into different intervals. When all the elements of this specimen were allocated into an interval, the total number of elements in each interval *E*Φ_*i*_ (Eq. (1)) was computed and the total area of each interval *A*Φ_*i*_ (Eq. (2)) was calculated from the individual area of each element. These values must fulfil the Eqs. (1) and (2). Then, the percentage area of each interval was computed in relation with the total area of the model for each specimen, following the Eq. (3).

(1) \begin{eqnarray*}& & \sum _{i=1}^{N}E{\Phi }_{i}=E=\text{Total number of Elements in the mesh}\end{eqnarray*}

(2) \begin{eqnarray*}& & \sum _{i=1}^{N}A{\Phi }_{i}=A=\text{Total Area of the Model}\end{eqnarray*}

(3) \begin{eqnarray*}& & A\Phi [\text{%}]= \frac{A{\Phi }_{i}}{\sum _{i}^{N}A{\Phi }_{i}} = \frac{A{\Phi }_{i}}{A} .\end{eqnarray*}

After carrying out this procedure, a new set of variables was generated (*A*Φ [%]), each one representing a different interval of stress values. Each variable represents the amount of area (as a percentage) of the original model having this range of stress values. If this procedure is performed in several models, these new variables can be used in later analyses comparing the different models.

A Fixed Upper Threshold *FT*_upper_ has to be defined in the interval Φ_*N*−1_ to allow including in the interval Φ_*N*_ all the highest values of stress. Some of these high values of stress represent artificial noise, which are numerical singularities produced in the results of FEA at the points where the displacement boundary conditions are applied (Marcé-Nogué et al., 2015b). The presence of this artificial noise in a numerical model is due to the mesh, since as the elements get smaller, the artificial high stress values become even higher (Dumont, Grosse & Slater, 2009). To select the *FT*_upper_ we chose not to consider the maximum value of 2% of the higher values of the model based in the suggestions of Walmsley et al. (2013) and Marcé-Nogué et al. (2016).

### Convergence of the data for the case study

To enable the comparison between different species it is necessary to specify the number of intervals that will be applied to the specimens under analysis, since this number will define the number of variables (*A*[%]). A large number of intervals will generate an excessive number of variables to work with, which can be difficult to interpret in later analyses. On the other hand, a limited number of intervals may yield incorrect results as they could oversimplify the stress pattern. Therefore, we decided to carry out multiple Principal Components Analyses (PCAs) as a guide to choose the minimum number of the intervals.

We performed these PCAs based on a different number of intervals to establish the threshold in which the data converged (i.e., when adding more intervals yielded similar patterns in the PCAs). We named this threshold value *N*_PCA_.

**Figure 2:**
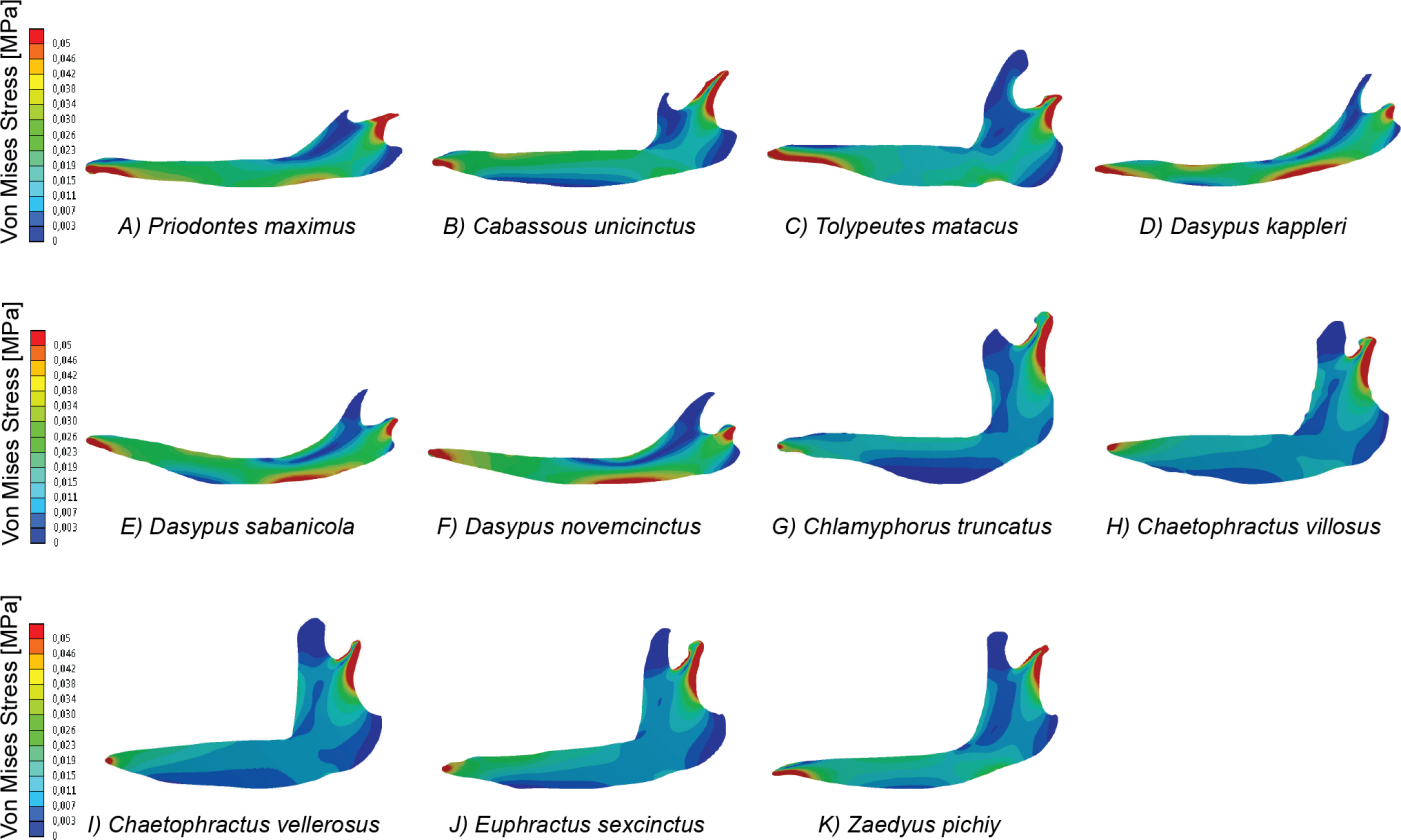
Map of von Mises stress distribution in the eleven FEA models of armadillo mandibles.

For the 11 mandible models of our case study (Fig. 2), the PCAs were performed using nine different sets of intervals: *N* = 3, 5, 10, 15, 20, 25, 50, 75 and 100. In this case the *FT*_upper_ was fixed to 0.1 MPa, which represents the appropriate value where less than the 2% of the higher values for all the eleven models are in its interval Φ_*N*_. Once all the PCAs were carried out we performed Major Axis Linear regressions between equivalent PC scores of one set of intervals and the next one (e.g., regression between the PC1 scores of the PCA of 3 intervals with PC1 scores of the PCA of 5 intervals). The coefficient of determination (*R*^2^) was then used to assess the convergence of the obtained results (Document S5). For each PC (1, 2 and 3 only, as they represent more than 80% of the variance for all the cases) we computed the coefficient of determination for successive pairs of PCs. The number of intervals at which *R*^2^ reached a plateau (i.e., it does not further increase) was considered the *N*_PCA_.

When the variables are all in the same units, it is usually preferred to carry out the PCA on the variance–covariance matrix. Since our variables were in the same units, we performed the aforementioned PCAs using the variance–covariance matrix. Nevertheless, if some variables, despite being in the same units, have a noticeably larger variance than others they will have a higher weight on the PCA, which might obscure more subtle relationships between those variables. In these kind of situations PCAs using the correlation matrix are more adequate (i.e., this is equivalent to perform the PCA using standardized variables). In the context of the present study this is relevant, since areas with high stress are usually small in all specimens. This implies that the variance for this specific interval (which represents one of the variables used to perform the PCA) will be small compared to the variance of an interval representing lower stress values, which occupy very large areas. Since the interval representing higher stresses could be still informative in comparative terms between species, it is important to account for this situation. By performing the PCA on the correlation matrix, a variable that has a smaller variance in absolute terms can be informative in relative terms. For this reason, in the case study we carried out PCAs using both kinds of variable matrices (i.e., variance–covariance and correlation).

## Results

### Validation of the data for statistical analyses

Document S4 show the percentage of area for each interval *A*Φ[%] using different *N* intervals, as well as the variance loadings for the PCAs.

**Figure 3:**
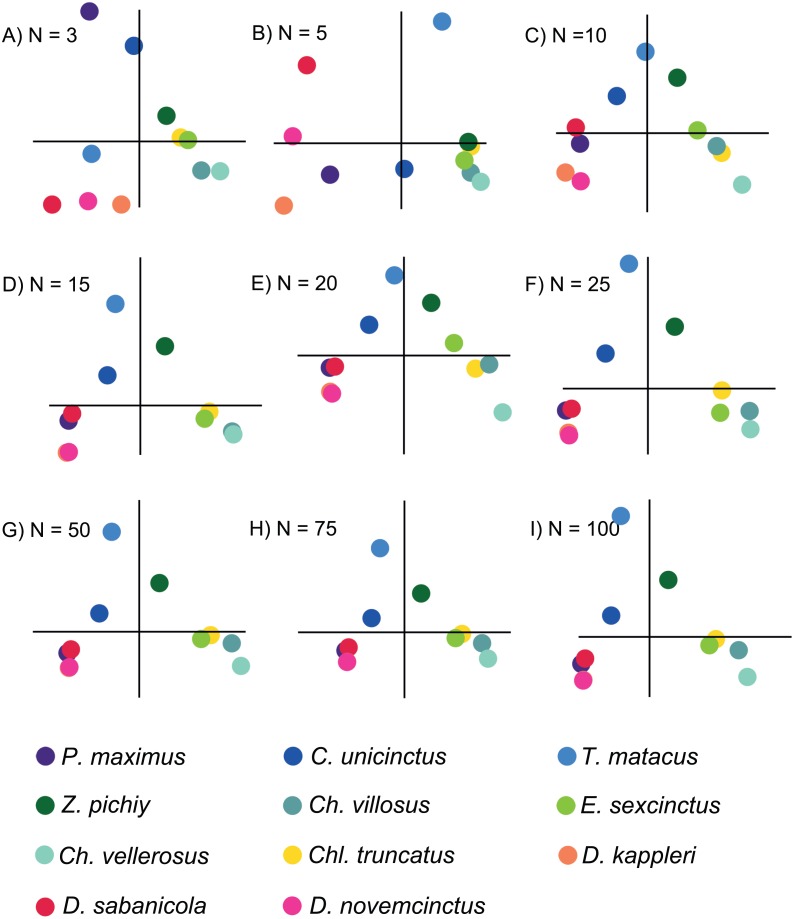
Plots displaying the first two PCs of the different PCAs for *N* = 3, 5, 10, 15, 20, 25, 50, 75 and 100. The species are coloured by subfamily: blue: Tolypeutinae, green: Euphractinae, red and pink: Dasyponinae, yellow: Clamyphorinae. The axes of each pair of PCs are in the same scale.

**Figure 4:**
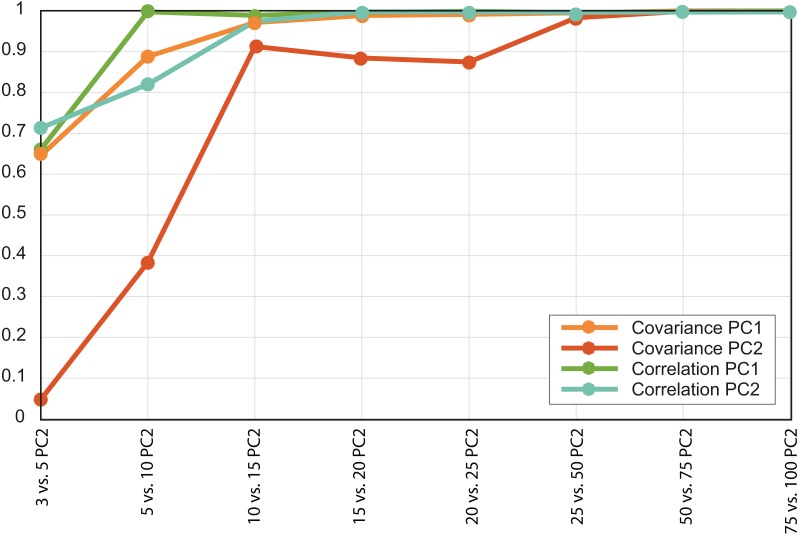
Convergence of the *R*^2^ values of the PC scores. Each value is the *R*^2^ for a different pair of PCAs, both the variance-covariance matrix based PCA (orange lines) and the correlation-matrix based PCAs (green lines). Each PC was correlated with the equivalent PC of the PCA developed using a larger number of intervals.

Based on the patterns observed in the plots displaying the first two PCs (Fig. 3) for each set of intervals it is clear that the distribution in stress space became more or less the same after *N* = 50. Additionally, the results of the regressions of the PCs indicate that the scores are almost completely correlated for *N* = 50 (Fig. 4 and Document S5), therefore we chose to compare the different species with the set of 50 intervals (i.e., *N*_PCA_).

### Case study

Using a *N*_PCA_ = 50, the PCA developed on the covariance matrix showed more than 90% of the variance concentrated in the first PC, while using the correlation matrix the first PC explained only 75.4% of the variance.

**Figure 5:**
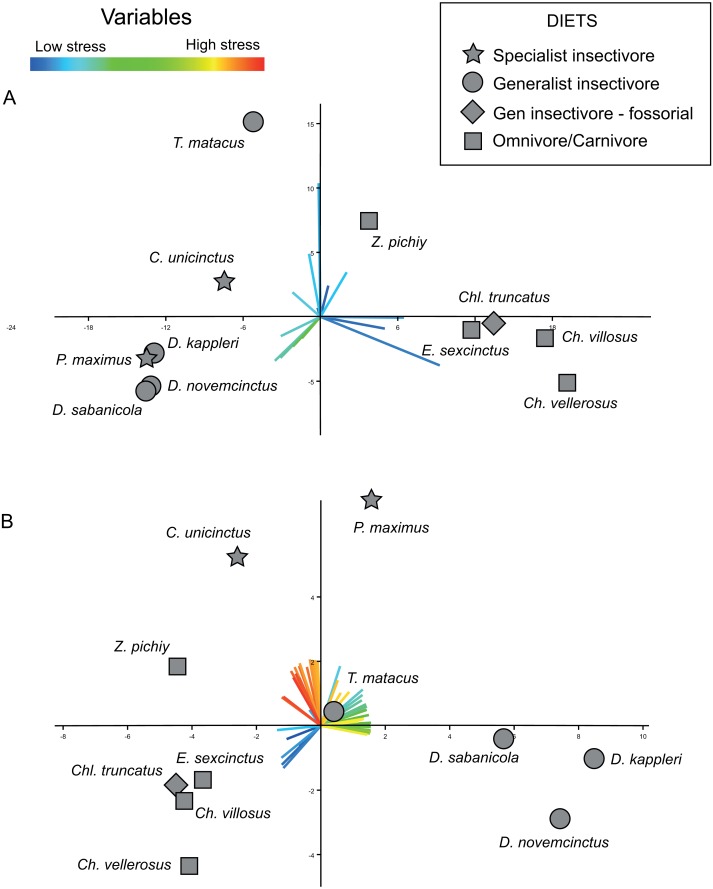
Plots displaying the two kinds of PCAs performed. (A) PCA based on the variance-covariance matrix. (B) PCA based on the correlation matrix. The loadings for each variable are coloured according with the range of stress they represent, with reddish colours for high level of stress, and bluish for low levels. *X*-axis: PC1. *Y*-axis: PC2.

The bi-plot of stress space defined for the first two PCs of the PCA performed on the variance–covariance matrix allow to identify different areas (Fig. 5A). The right side of this bi-plot (Fig. 5A) is occupied by species that can be described as having a very large area of a specific range of values (i.e., low to very low stress), whilst downwards on the left of this stress space are specimens characterized by moderate stress values. Finally, the upper left part of this stress space is exclusively occupied by individuals showing values intermediate between the former ones. Based on the loadings of the variables, is evident that the proportionally larger areas with low stress dominate this PCA (Fig. 5B).

The results obtained from this PCA make it possible to establish certain patterns. A proportionally larger area of intermediate stresses characterizes the insectivores, while proportionally larger areas with very low stress values characterize the omnivores (Fig. 5A). However, within this trend two species showed a distinct pattern in PC2: *T. matacus* and *Z. pichiy*. Both are located in the upper part of the plot (Fig. 5A), characterized mainly by one specific value of low stress area. Nonetheless, the second PC only explains 16.8% of the variance.

According to a hypothesis suggested in Serrano-Fochs et al. (2015), high levels of stress along the mandible would represent a fragile mandible with a reduced capability to chew or process hard items. Supporting this hypothesis, in the present case the insectivores are in the left part of the stress space, defined by intermediate to low stress levels. This can be expected considering that these species feed mainly on ants without chewing them; thus their mandibles are expected to show a higher stress level. On the other hand, very low stress values would be indicative of mandible with a higher ability to bear higher loads. This would be in agreement with the location of the omnivorous taxa at the right side of the stress space characterized by intermediate stress values (*E. sexcintus, Ch. villosus, Ch. vellerosus* and *Z. pichiy*, Fig. 5A).

Nevertheless, this PCA did not detect any difference in the stress pattern between specialist and generalist insectivores. All the loadings for the first two PCs belong to low or intermediate stress values, whilst the loadings of the variables representing intermediate to high stress have almost negligible values. This arises because the percentage of areas with high values is small in all analysed mandibles (the variables representing intermediate and high stresses have very small loadings for PC1 and PC2). Since we carried out the PCA using the variance–covariance matrix, the larger absolute values of the lower stress areas implied larger variance for those variables, thus completely masking the variability that might exists in the variables representing high stress values.

In the case of the PCA using the correlation matrix the distribution of the loadings of the variables (Fig. 5B) is noticeably more homogeneous (more variables have an important contribution to the PCs analysed) without being so strongly influenced by just a few specific variables as was the case of the PCA performed using the variance–covariance matrix (where variables representing low stress values overcome the rest of the variance). The PCA of the correlation matrix successfully distinguishes the main three diets, with omnivore-carnivore species on lower-left area of the plot. PC2 separates specialist insectivores from the rest of species, while *Chlamyphorus truncatus* (i.e., a generalist insectivorous species exhibiting a very particular diet due to its completely fossorial life style) is located near the omnivore/carnivore species in the negative part of PC1 and PC2.

Within this PCA, omnivore/carnivore species were characterized by very low stress values (lower-left quadrant of the plot), whilst generalist insectivores showed a proportionally larger area of intermediate stress values than the rest of the species. Specialist insectivores have proportionally larger areas of high stress.

As was the case when performing the PCA using the variance–covariance matrix, the fossorial generalist insectivore—*Chlamyphorus truncatus* was located with the omnivores. In spite of the fact that insects correspond to large part of its diet, this species also feeds on worms, snails, and a small proportion of roots (Borghi et al., 2011). This consumption of relatively harder objects that need more oral processing than just tiny insects, might explain the biomechanical results obtained here.

As similarly found by the variance–covariance PCA, *Z. pichiy* corresponds to the omnivorous species with proportionally less area of low stress. This species is considered to show a preference for soft-bodied species (e.g., larvae, tarantula spiders, pods) (Redford, 1985), thus being the least carnivorous taxon among all the omnivore/carnivore armadillos (Superina, Pagnutti & Abba, 2014). Indeed, microwear studies classified *Z. pichiy* together with the insectivorous species (Green, 2009). In concordance with this information, our results show that *Z. pichiy* is located in the areas of the stress space between carnivore and insectivore species. Finally, it has been suggested that *T. matacus* has a seasonal diet based on fruits and pods representing more than 50% of its consumed items during the dry season (Bolkovi, Caziani & Protomastro, 1995), being the the generalist insectivores species showing the lowest stress.

## Discussion

### Influence of the mesh in the results

The methodology presented in this work is based on obtaining the percentage areas containing certain values of stress. To validate the results, the influence of coarse meshes and non-homogeneous meshes on the computed areas was also tested in the Supplementary Information, where methods and results are discussed (Document S1).

According to the results obtained (Figs. S5A and S5B and Tables S1 and S2, our proposed approach is not affected by the size of the elements (i.e., we obtained extremely low values of the relative error ($REr{r}_{i}^{j}$)). In addition, when the homogeneity of the mesh was tested, the absolute error (*AErr*^*j*^) and its variance showed very low values as well Figs. S5C and S5D and Tables S3 and S4, hence confirming that the homogeneity of the mesh is not affecting the newly generated variables.

Overall, this means that under our proposed methodology the size and the homogeneity of the mesh do not affect the obtained results, thus providing a robust method that is effective irrespective of these factors. Therefore, it is not necessary to apply mesh correction techniques such as generating a Quasi-Ideal Mesh (QIM) as proposed by Marcé-Nogué et al. (2016).

### Convergence of the data in the case study; which *N* is adequate?

There were important differences between the PCAs depending on the *N* value. When this value was low (i.e., *N* = 3 and 5) the obtained results were noticeably different, whereas from *N* = 15 and above the PCA results were very similar, as shown by the dispersion plots of the first and second PCs (Fig. 4).

In the case study, the PCA convergence was *N*_PCA_ = 50. This means that at least for structures such as armadillo jaws, 50 intervals are enough to describe the sample variability in terms of distribution of stress values per area. In fact, for the purpose of this analysis (i.e., to distinguish between dietary groups) around 15 intervals would have been enough to describe at least the main eco-morphological categories.

Whether *N* = 50 is an adequate number of intervals for other cases is something that needs to be addressed in the future and that might depend on the structure and sample under analysis. It is likely that for more complex geometries or if species from other taxonomic groups are included, the *N*_PCA_ will differ. Based on our present results, we suggest that the *N*_PCA_ should be determined on a case-by-case basis.

### Comparative biomechanical performance of extant armadillos

Using a *N*_PCA_ = 50, the PCA of the variance–covariance matrix showed differences between omnivores and both groups of insectivores (Fig. 5A). These observed differences are concordant with previously published results (Serrano-Fochs et al., 2015; Marcé-Nogué et al., 2016), although the present results provide a better distinction of the groups when compared to similar study that only analysed the stress values collected at specific points (Serrano-Fochs et al., 2015) (Fig. 5A). The mandibular ecomorphology of Cingulata has been previously studied using morphometrics, microwear and biomechanics, among other techniques (Fariña, 1985; Fariña, 1988; Vizcaíno & Fariña, 1997; Vizcaíno & Bargo, 1998; Vizcaíno, De Iuliis & Bargo, 1998; De Iuliis, Bargo & Vizcaíno, 2000; Fariña & Vizcaíno, 2001; Vizcaíno et al., 2004; Vizcaíno, 2009; De Esteban-Trivigno, 2011). Nevertheless, the results obtained applying our proposed approach are more accurate when distinguishing between dietary groups. It is noteworthy that none of the previously proposed methods to compare mandibular performance using FEA applied to different species of armadillos (Serrano-Fochs et al., 2015; Marcé-Nogué et al., 2016) were able to effectively distinguish the specialist from the generalist insectivores. Consequently, the approach presented here, especially when using the correlation matrix in the PCA, seems to be able to detect subtler differences, thus representing a useful way to characterize and understand the biomechanical behaviour of the mandibles in relation to diet.

The bi-plot of the first two PCs of the PCA computed using the correlation matrix displayed *T. matacus* and *Z. pichiy* in a location that was slightly different than the rest of species of their corresponding dietary group. Classifying diets is always a complicated matter, as it is reducing the existing dietary variability to only a few categories. As explained in the results section, these two species have a diet that differs from the general trend of their groups. In a similar fashion, *Chl. truncatus* occupies a position along the omnivore/carnivore group instead of showing stress values similar to other insectivores. As previously mentioned, even though this species has been usually grouped with insectivores, *Chl. truncatus* feeds on worms, snails and roots; all items that need more processing if compared to simply swallowing small insects. It is also relevant that the PCAs of the stress intervals were able to detect these differences in diet, including some subtle differences that have been elusive using other techniques. Even though further studies are required, the proposed procedure seems to be a promising approach to be tested in future ecomorphological studies considering other taxonomic groups.

The PCA computed using the correlation matrix provides information on the variability of all the variables, giving them all the same importance through standardization. This explains why it was possible to distinguish between the two insectivore’s diets even if their differences were rather subtle (Fig. 5B). Nonetheless, interpreting the loadings from the correlation-based PCA can be misleading if it is not properly done. The loadings of the PCA performed using the variance–covariance matrix can be easily interpreted; in the present study, a variable (*A*Φ[%]) with a higher loading for a given PC represents a larger actual area for that interval of stress values in the mandibles of the specimens located in the direction of that loading. However, in the PCA carried out using the correlation matrix (i.e., where the variables have been standardized), a variable with a higher loading indicates that this variable has a proportionally larger variability in that area of the stress space (but not necessarily in absolute terms). For example, in the Fig. 5B *C. unicintus* is located in region of the stress space that corresponds to higher loads for high and very high stress variables. This does not mean that the species in question has an area with high stresses larger than its area with small stresses in absolute terms. Rather, it indicates that these high stress variables are proportionally larger as compared with the rest of species. It is important to bear this in mind when using correlation matrix-based PCAs to interpret the results obtained from the intervals method proposed here. As discussed above, overall both PCAs (i.e., using the variance–covariance or correlations matrices) have shown to be very useful for identifying ecomorphological patterns based on stress values obtained from FEA.

We have successfully reduced thousands of data points to just 50 variables that are still biologically meaningful. In addition, we managed to use the output data from FEA, which has a different number of data for each specimen (i.e., due to the differences in the number of elements in each model) and represent it with the same 50 variables in each specimen under analysis making them comparable in a statistical framework. We have shown that at least for our case study this approach works better than previously published examples which are based on either the visual inspection of the stress maps, collecting stress data on specific points, or comparing the complete stress value distributions (Serrano-Fochs et al., 2015; Marcé-Nogué et al., 2016). We consider that for comparative analysis where the main aim is to test the same functional hypothesis under equivalent loads in different species, this method can be generalized to other taxonomic groups and structures. Additionally, it is promising that in addition to the PCAs other techniques might be applied using this newly generated stress variables (intervals) depending on the sample and the biological hypotheses being tested (a matter that should be explored in future studies).

Although the proposed approach implies losing the spatial information of the stress, the case study analysed here strongly supports that the new interval variables are still biologically meaningful and easily interpretable, thus being very useful to describe and analyse the data. Our proposed approach is especially appropriate in cases where the amount of stress might vary but the stress spatial distribution is highly similar. In these situations, it is very difficult to interpret the obtained results, as well as to answer the tested biological hypothesis just by visually inspecting the coloured stress maps. It is not clear yet whether this will work or not in cases where the analysed structures are very different (e.g., when comparing dissimilar morphologies at a higher taxonomic level where usually anatomical differences tend to be prominent). For this reason, we recommend that the newly proposed intervals method be used together with other traditional approaches, such as the visual inspection of the stress and/or deformation maps.

Further research is required to test the advantages and limitations of the intervals method when analysing other anatomical structures and/or taxonomic groups, as well as testing it using three-dimensional models (where the same method can be applied just by simply using volume instead of area in the equations presented here).

## Conclusions

The methodology proposed in this work has shown to be applicable independently of the characteristics of the mesh. This is a really useful feature of the method, since most meshes representing biological objects are non-uniform. In addition, the proposed intervals method allows the quantitative comparison of FEA results in a multivariate framework.

Furthermore, when applying this method to our biological case study of armadillo mandibular performance, we have shown that it is a powerful tool to characterize the biomechanical behaviour of the mandibles in relation to different dietary groups, allowing the distinction between different diets, including discrimination between specialist and generalist insectivore species.

This new methodology should be tested in other taxa, as well as at higher taxonomic levels where the stress distribution might be more dissimilar between different taxa. Nonetheless, the positive results obtained when analysing a case study known for its difficulty such as the armadillos suggests that the proposed approach is promising and represents a useful method to be included in the FEA toolkit for comparative analyses.

##  Supplemental Information

10.7717/peerj.3793/supp-1 Supplemental Information 1 Document S1 Convergence of the mesh.

10.7717/peerj.3793/supp-2 Supplemental Information 2 Document S2 Influence of the size of the mesh.

10.7717/peerj.3793/supp-3 Supplemental Information 3 Document S3 Influence of the homogeneity of the mesh.

10.7717/peerj.3793/supp-4 Supplemental Information 4 Document S4 Intervals for the armadillos.

10.7717/peerj.3793/supp-5 Supplemental Information 5 Document S5 Scores of different intervals.

## Additional Information and Declarations

### Data Availability

The following information was supplied regarding data availability: The raw data has been supplied as a Supplementary File.
