# Supplementary material for: The intervals method: a new approach to analyse finite element outputs using multivariate statistics
Source: PeerJ. 2017 Oct 13;5:e3793. doi: 10.7717/peerj.3793 (PMC5642245; doi:10.7717/peerj.3793)
Supplement: Supplemental Information 1 — Convergence of the mesh. [file peerj-05-3793-s001.doc]

**SUPPLEMENTARY INFORMATION**

**The intervals method: A new approach to analyse finite element outputs using multivariate statistics.**

Jordi Marcé-Nogué, Soledad de Esteban-Trivigno, Thomas A. Püschel and Josep Fortuny

# Testing the influence of the size of the mesh in the results

To test the mesh influence in the results of our proposed approach, twelve different meshes of a *Chlamyphorus truncatus* mandible were created with the set of forces and boundary conditions described above. Each one was meshed with different overall size element, generating finer meshes each time. In Finite Element mesh generation, this overall size element does not mean that all the elements have same size. It means that the elements, whenever it is geometrically possible, have approximately similar size.

Although previous works studied the influence of the size of the elements of the mesh in the stress and strain results (Bright & Rayfield, 2011; Tseng & Flynn, 2014), here we studied how the change in the size of the elements of the mesh affects the values of the areas we are computing in this new methodology.

Considering these meshes (figure S1 of the supplementary information), different analyses were run on each different set of number of intervals (being N the number of intervals, for N=3, 5, 10, 15, 20, 25, 50, 75 and 100; and *i* each one of the intervals for each N). In all the analyses, FT_upper_ was fixed to 0.1 MPa, a suitable value because less than the 2% of the high values are included in the interval Φ_N_ (see Table S1 and Table 2). These meshes were used previously in (Marcé-Nogué et al., 2016) where are fully described.

When AΦ_i_[%] were calculated for all the twelve meshes (each mesh was defined by *j*), it could be assumed that Mesh j=12 is the Quasi-Ideal Mesh (QIM) and the relative error of each mesh
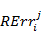
could be calculated in function of this QIM as follow


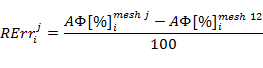


At this point we had N relative Errors for each mesh *j*. To have an overall Absolute Error for each mesh, the average and the variance of the Absolute Error was computed as follow:


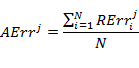


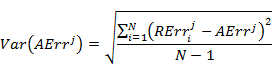


After performing the FEA and calculating the Von Mises Stress values (Figure S3), absolute errors AErr^j^ were obtained for the twelve meshes and for the different values of N (intervals) studied. Most of the errors are smaller than 2% (Figure S5(a) and S5(b) and Table S1). However, when studying the variance of the Absolute Error, very coarse meshes (j=1 and j=2) presented substantial values of dispersion, whereas coarse meshes and fine meshes (up to j=3) presented small amount of dispersion (Table S2).

Document S2 of the supplementary information compiles the percent of area of each interval AΦ[%] and the relative error of each mesh
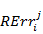
for the j=12 meshes and the different N values herein analysed.

# Testing the influence of the homogeneity of the mesh in the results

Six different meshes of a *Chlamyphorus truncatus* mandible were tested with the set of forces and boundary conditions described above. In this case, the meshes were grading from an absolute non-uniformity mesh to a quasi-uniform mesh. As in the previous case, the complexity of the geometry makes a perfect uniform mesh impossible.

For the meshes of figure S2, the same analysis than in the previous section were done on the same interval subdivisions (for N=3, 5, 10, 15, 20, 25, 50, 75 and 100). In all the analysis, FT_upper_ was fixed to 0.1 MPa, a suitable value because less than the 2% of the high values are in the interval Φ_N_ (Table S3 and Table S4). These meshes were used previously in (Marcé-Nogué et al., 2016), where they are fully described.

Von Mises Stress distribution was obtained (Figure S4) for the six different meshes. The non-homogeneity of the mesh did not affected the results. Most values obtained for the six meshes and for the different values of N analysed were below 1% for the absolute error AErr^j^ and below 2% for the variance of this error (Figure S5(c) and S5(d) and Tables S2 and S4). The percentual area for each interval AΦ[%] and the relative error of each mesh
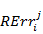
 for the j=6 meshes as well as the different N values analysed herein can be found in document S3 of the supplementary information.

**Table S1: Absolute Error** **AErr^j^** obtained for the j=12 meshes analyzed and for the different values of N studied when evaluating the influence of size of the elements in the mesh.

|  | **N=3** | **N=5** | **N=10** | **N=15** | **N=20** | **N=25** | **N=50** | **N=75** | **N=100** |
| --- | --- | --- | --- | --- | --- | --- | --- | --- | --- |
| **j=1** | 0.5217 | 0.3300 | 2.0642 | 1.7437 | 1.9937 | 1.5769 | 0.9060 | 0.6874 | 0.5851 |
| **j=2** | 0.5435 | 0.3425 | 0.4125 | 1.1330 | 0.9648 | 0.5842 | 0.6562 | 0.4569 | 0.4215 |
| **j=3** | 0.2226 | 0.1335 | 0.7344 | 0.6935 | 0.2793 | 0.3861 | 0.4566 | 0.4270 | 0.2921 |
| **j=4** | 0.4678 | 0.3451 | 0.2155 | 0.1863 | 0.3265 | 0.3560 | 0.2835 | 0.2600 | 0.2718 |
| **j=5** | 0.1725 | 0.2283 | 0.3234 | 0.3432 | 0.1649 | 0.2780 | 0.1867 | 0.2576 | 0.1599 |
| **j=6** | 0.1082 | 0.1055 | 0.1443 | 0.0724 | 0.0764 | 0.0900 | 0.0917 | 0.0817 | 0.0772 |
| **j=7** | 0.0552 | 0.0536 | 0.1095 | 0.0723 | 0.0529 | 0.0566 | 0.0468 | 0.0364 | 0.0361 |
| **j=8** | 0.0233 | 0.0331 | 0.0672 | 0.0567 | 0.0542 | 0.0510 | 0.0384 | 0.0319 | 0.0263 |
| **j=9** | 0.0290 | 0.0324 | 0.0486 | 0.0405 | 0.0288 | 0.0337 | 0.0240 | 0.0207 | 0.0190 |
| **j=10** | 0.0106 | 0.0101 | 0.0177 | 0.0133 | 0.0098 | 0.0092 | 0.0100 | 0.0084 | 0.0070 |
| **j=11** | 0.0049 | 0.0041 | 0.0087 | 0.0085 | 0.0072 | 0.0076 | 0.0055 | 0.0053 | 0.0046 |
| **j=12** | 0.0000 | 0.0000 | 0.0000 | 0.0000 | 0.0000 | 0.0000 | 0.0000 | 0.0000 | 0.0000 |

**Table S2: Variance of the** **Absolute Error AErr^j^** obtained for the j=12 meshes analyzed and for the different values of N studied when evaluating the influence of size of the elements in the mesh.

|  | **N=3** | **N=5** | **N=10** | **N=15** | **N=20** | **N=25** | **N=50** | **N=75** | **N=100** |
| --- | --- | --- | --- | --- | --- | --- | --- | --- | --- |
| **j=1** | 0.0528 | 0.1043 | 11.5771 | 10.7048 | 18.4257 | 8.3023 | 3.9476 | 2.5524 | 2.2136 |
| **j=2** | 0.0606 | 0.0876 | 0.1036 | 2.8865 | 2.7587 | 0.5606 | 1.5156 | 0.6008 | 0.5208 |
| **j=3** | 0.0165 | 0.0146 | 1.0689 | 1.4602 | 0.0585 | 0.2796 | 0.5935 | 0.7742 | 0.3718 |
| **j=4** | 0.0668 | 0.0438 | 0.0199 | 0.0128 | 0.1491 | 0.2218 | 0.1159 | 0.2029 | 0.2236 |
| **j=5** | 0.0153 | 0.0285 | 0.0382 | 0.1173 | 0.0229 | 0.0752 | 0.0517 | 0.1504 | 0.0740 |
| **j=6** | 0.0065 | 0.0097 | 0.0155 | 0.0077 | 0.0060 | 0.0115 | 0.0115 | 0.0108 | 0.0122 |
| **j=7** | 0.0006 | 0.0018 | 0.0279 | 0.0094 | 0.0069 | 0.0054 | 0.0031 | 0.0025 | 0.0036 |
| **j=8** | 0.0001 | 0.0007 | 0.0070 | 0.0077 | 0.0075 | 0.0060 | 0.0030 | 0.0021 | 0.0025 |
| **j=9** | 0.0002 | 0.0005 | 0.0041 | 0.0035 | 0.0026 | 0.0022 | 0.0010 | 0.0009 | 0.0009 |
| **j=10** | 0.0000 | 0.0001 | 0.0007 | 0.0005 | 0.0003 | 0.0003 | 0.0001 | 0.0001 | 0.0001 |
| **j=11** | 0.0000 | 0.0000 | 0.0001 | 0.0001 | 0.0001 | 0.0001 | 0.0000 | 0.0000 | 0.0000 |
| **j=12** | 0.0000 | 0.0000 | 0.0000 | 0.0000 | 0.0000 | 0.0000 | 0.0000 | 0.0000 | 0.0000 |

**Table S3: Absolute Error AErr^j^** obtained for the j=6 meshes analyzed and for the different values of N studied when evaluating the influence of the homogeneity of the mesh.

|  | **N=3** | **N=5** | **N=10** | **N=15** | **N=20** | **N=25** | **N=50** | **N=75** | **N=100** |
| --- | --- | --- | --- | --- | --- | --- | --- | --- | --- |
| **j=1** | 0.2108 | 0.1265 | 0.7054 | 0.6778 | 0.2557 | 0.3683 | 0.4312 | 0.4288 | 0.2845 |
| **j=2** | 0.5474 | 0.3982 | 0.4206 | 0.3742 | 0.2850 | 0.2758 | 0.2965 | 0.3326 | 0.2727 |
| **j=3** | 0.4887 | 0.4204 | 0.4979 | 0.2609 | 0.2364 | 0.2248 | 0.1709 | 0.1765 | 0.1918 |
| **j=4** | 0.2456 | 0.1473 | 0.3422 | 0.1868 | 0.1790 | 0.2577 | 0.2449 | 0.1971 | 0.1425 |
| **j=5** | 0.0108 | 0.0117 | 0.0268 | 0.0290 | 0.0295 | 0.0275 | 0.0408 | 0.0274 | 0.0334 |
| **j=6** | 0.0000 | 0.0000 | 0.0000 | 0.0000 | 0.0000 | 0.0000 | 0.0000 | 0.0000 | 0.0000 |

**Table S4: Variance of the Absolute Error AErr^j^** obtained for the j=6 meshes analyzed and for the different values of N studied when evaluating the influence of the homogeneity of the mesh.

| **mesh** | **N=3** | **N=5** | **N=10** | **N=15** | **N=20** | **N=25** | **N=50** | **N=75** | **N=100** |
| --- | --- | --- | --- | --- | --- | --- | --- | --- | --- |
| **j=1** | 0.0109 | 0.0069 | 0.9377 | 1.3502 | 0.0376 | 0.2266 | 0.5337 | 0.8224 | 0.3767 |
| **j=2** | 0.1018 | 0.0909 | 0.1803 | 0.1656 | 0.0912 | 0.0886 | 0.1240 | 0.2773 | 0.2255 |
| **j=3** | 0.0769 | 0.0695 | 0.1911 | 0.0741 | 0.0349 | 0.0245 | 0.0197 | 0.0332 | 0.0743 |
| **j=4** | 0.0238 | 0.0063 | 0.0977 | 0.0184 | 0.0204 | 0.0464 | 0.1154 | 0.0713 | 0.0313 |
| **j=5** | 0.0000 | 0.0000 | 0.0004 | 0.0006 | 0.0009 | 0.0004 | 0.0029 | 0.0011 | 0.0022 |
| **j=6** | 0.0000 | 0.0000 | 0.0000 | 0.0000 | 0.0000 | 0.0000 | 0.0000 | 0.0000 | 0.0000 |

**Figure S1 - Twelve meshes of *Chlamyphorus truncatus*** used to evaluate the influence of size of the elements in the mesh. Meshes generated from a very coarse one (Mesh 1) to a much fined one (Mesh 12).


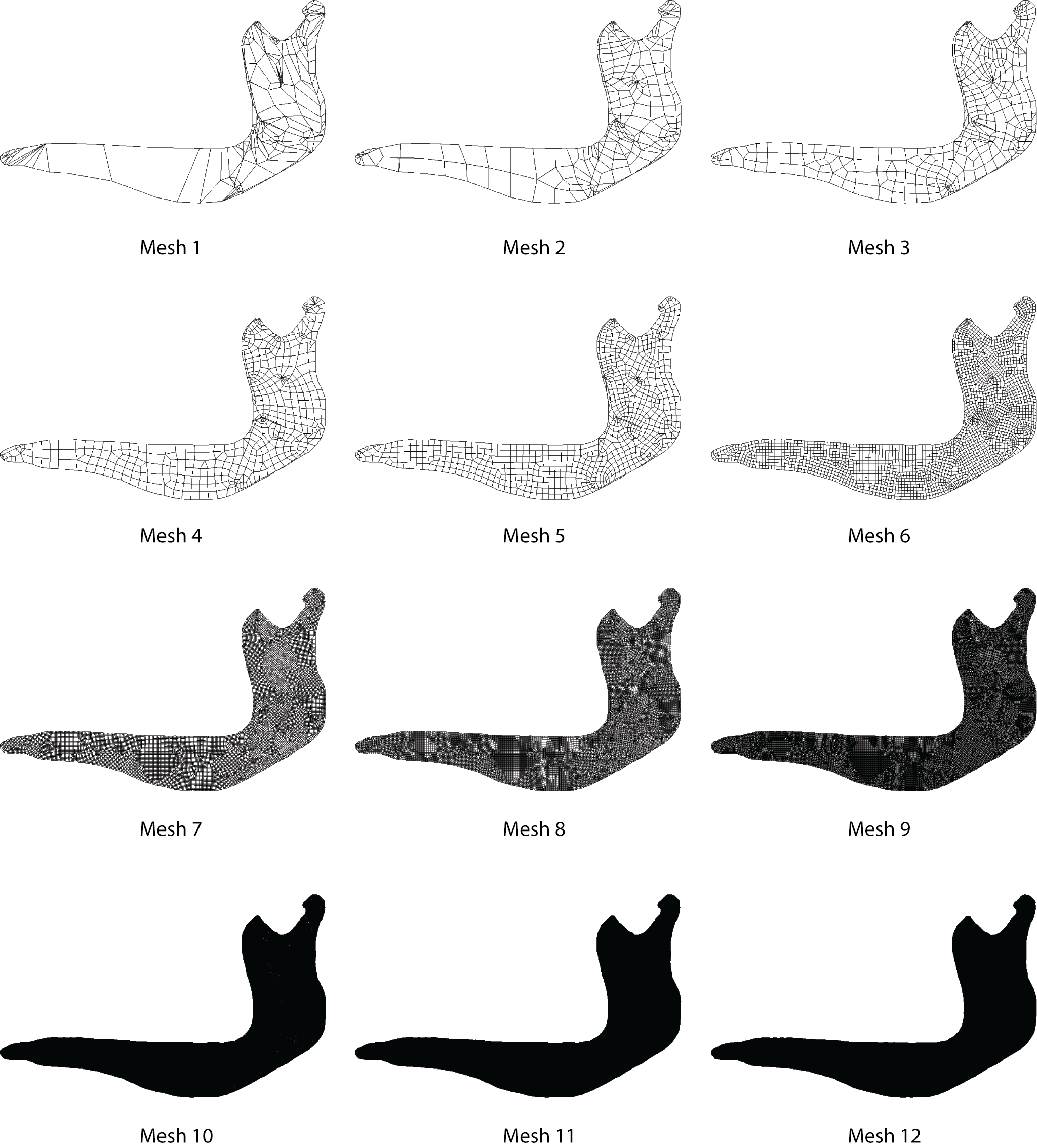


**Figure S2 - Six meshes of *Chlamyphorus truncatus*** used when evaluating the influence of the homogeneity of the mesh. Note the differences from an absolute non-uniform mesh (Mesh 1) to a quasi-uniform mesh (Mesh 6).


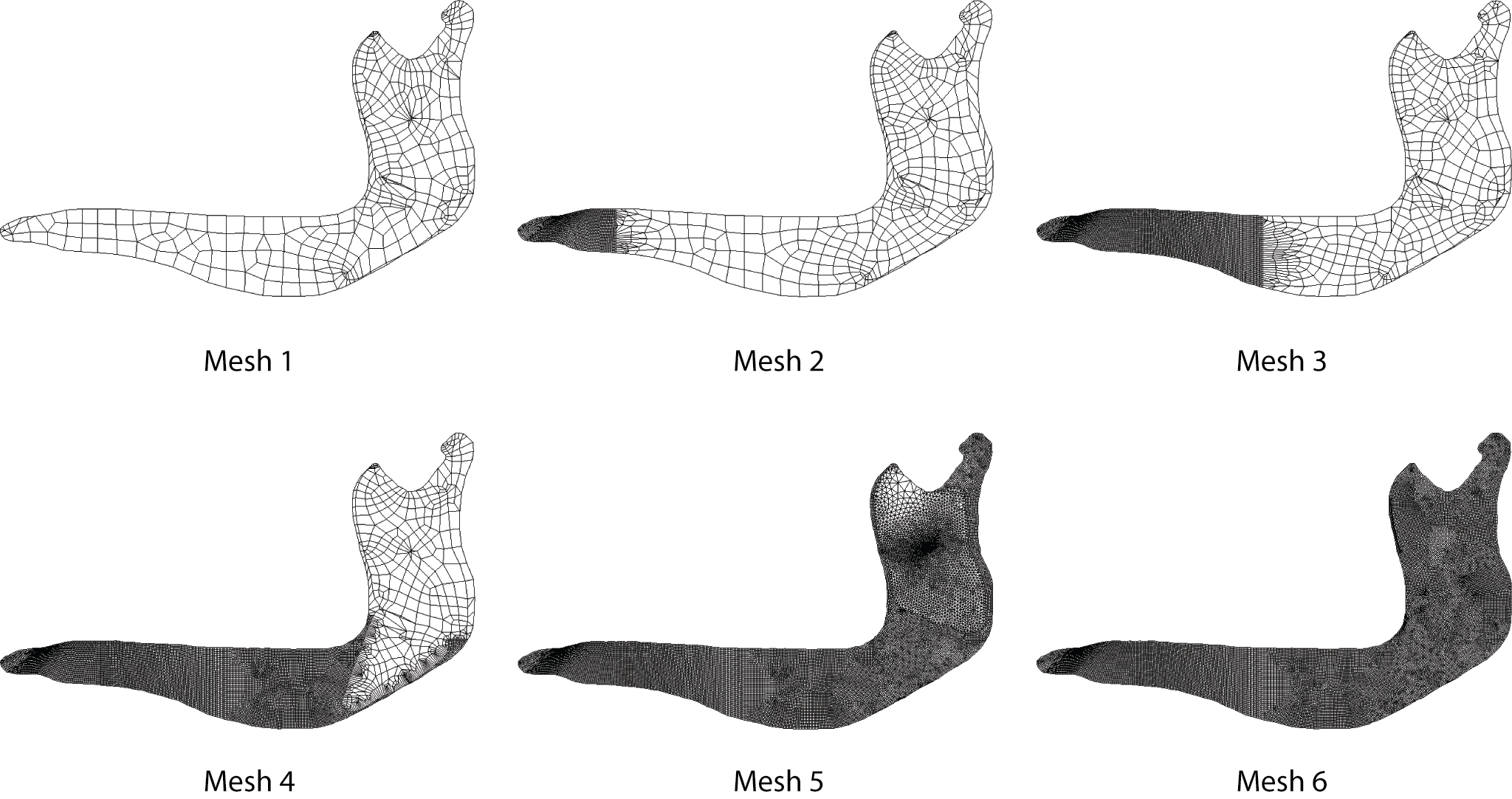


**Figure S3 - Map of Von Mises stress distribution** in the twelve meshes of *Chlamyphorus truncates* obtained when evaluating the influence of the size’s elements in the mesh.


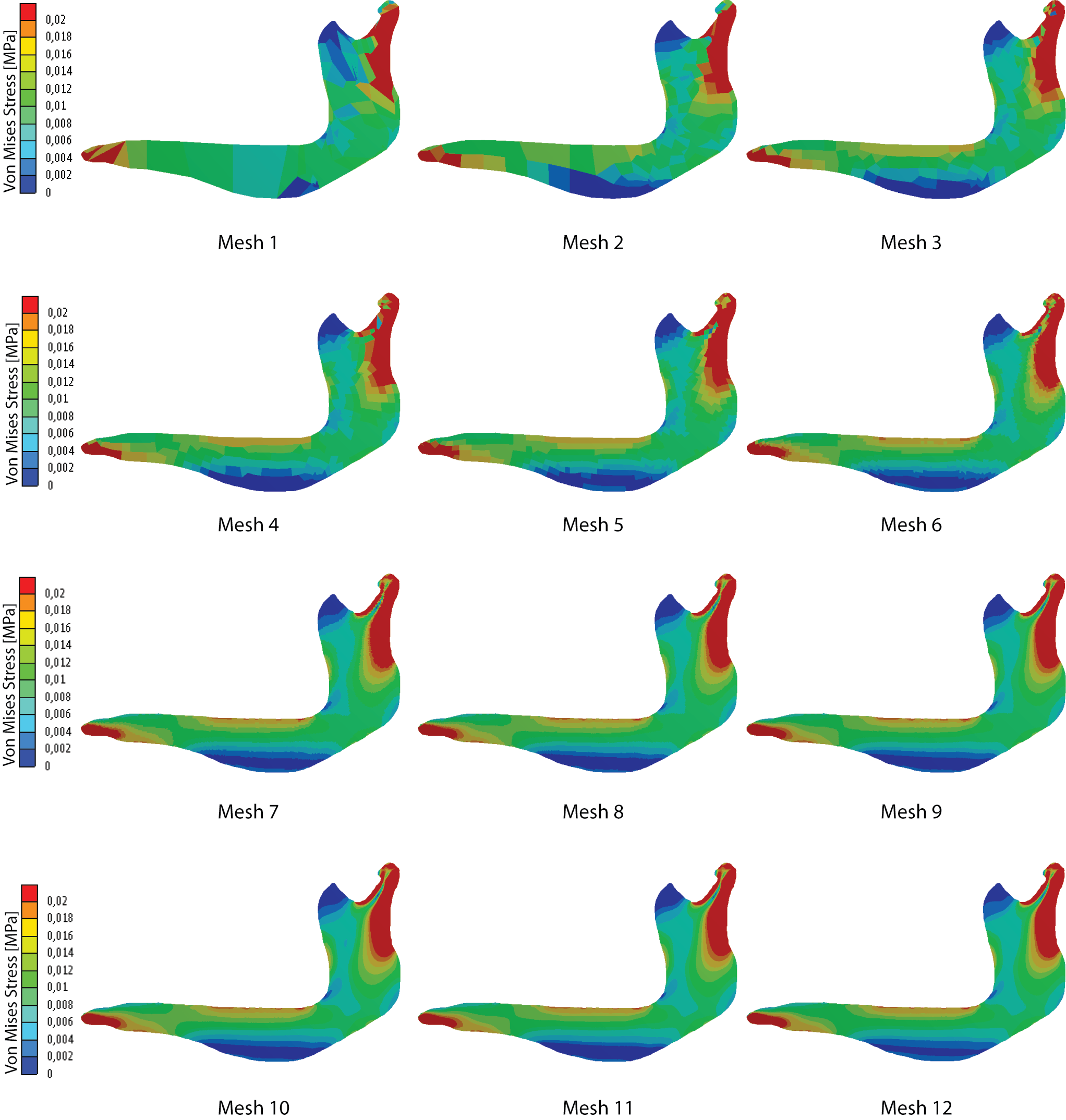


**Figure S4 - Map of Von Mises stress distribution** in the six meshes of *Chlamyphorus truncates* obtained when evaluating the influence of the homogeneity of the mesh.


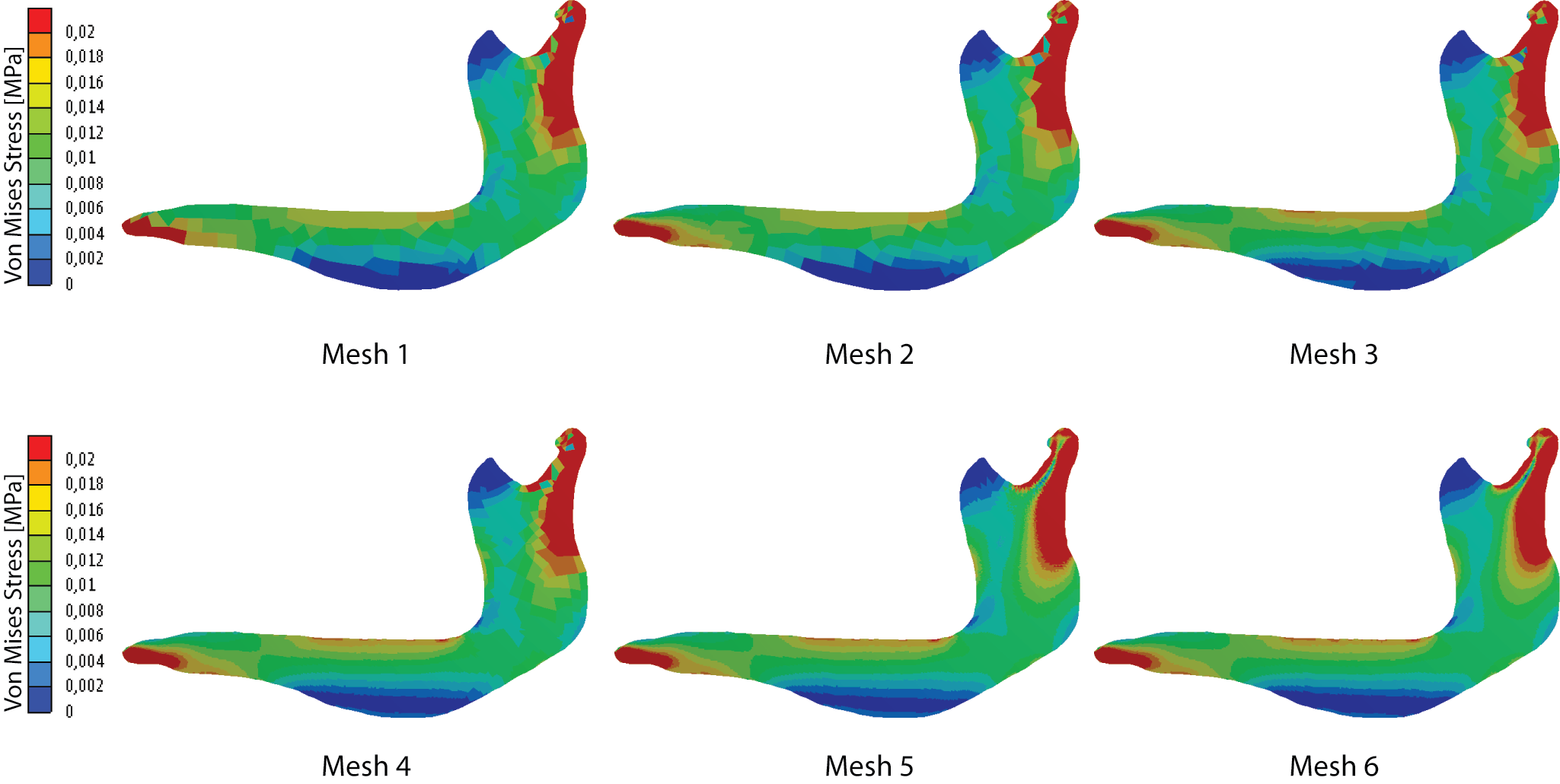


**Figure S5 - Evolution of the Absolute Error AErr^j^ and the variance** for the different values of N when (a), (b) testing the influence of the size of the mesh in the results and (c), (d) testing the influence of the homogeneity of the mesh in the results.


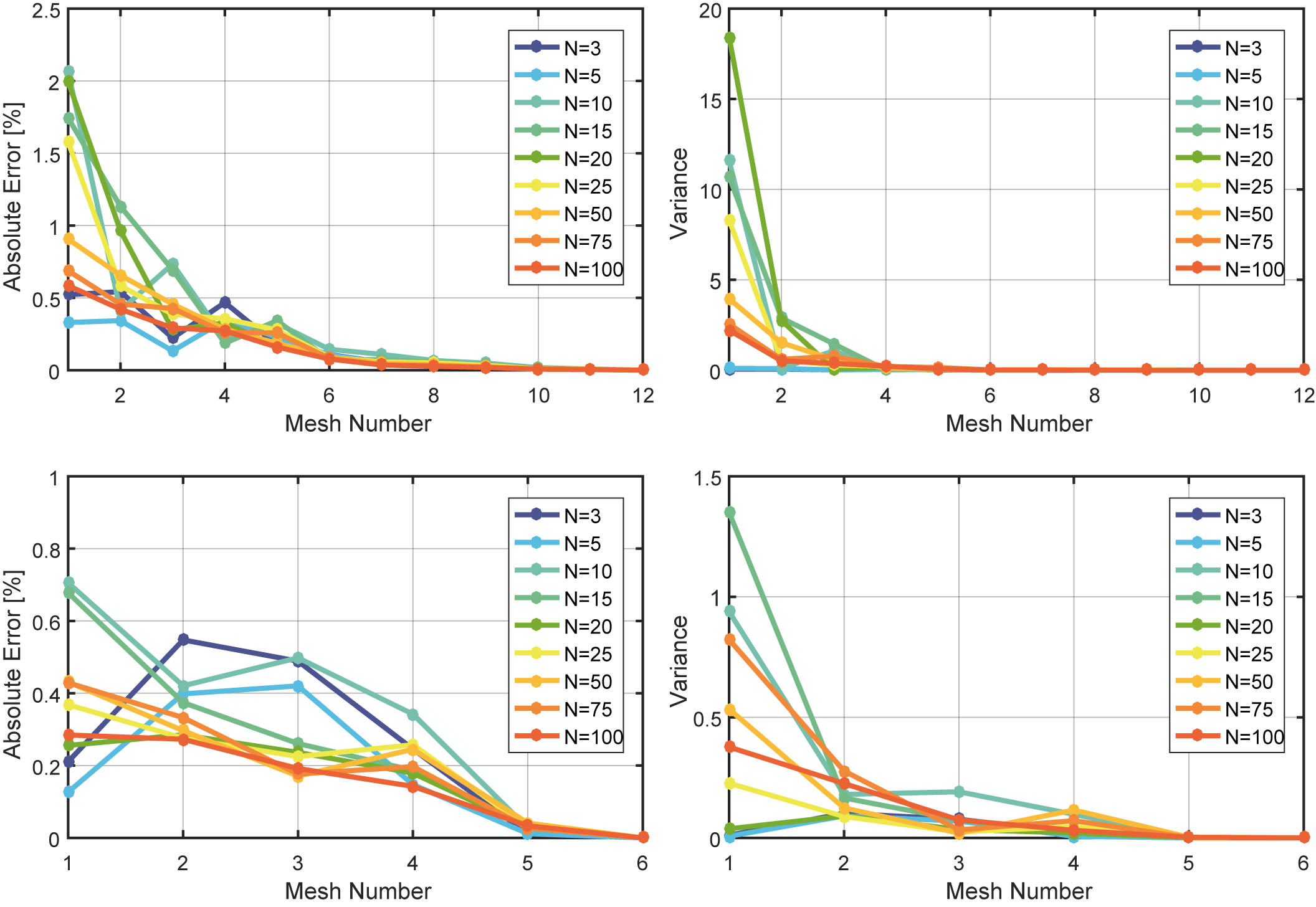


**References**

Bright JA., Rayfield EJ. 2011. The response of cranial biomechanical finite element models to variations in mesh density. *Anatomical record (Hoboken, N.J. : 2007)* 294:610–20. DOI: 10.1002/ar.21358.

Marcé-Nogué J., De Esteban-Trivigno S., Escrig C., Gil L. 2016. Accounting for differences in element size and homogeneity when comparing Finite Element models: Armadillos as a case study. *Palaeontologia Electronica* 19:1–22.

Tseng ZJ., Flynn JJ. 2014. Convergence analysis of a finite element skull model of Herpestes javanicus (Carnivora, Mammalia): implications for robust comparative inferences of biomechanical function. *Journal of Theoretical Biology* 365:112–148. DOI: 10.1016/j.jtbi.2014.10.002.
